# Asynchronous Replication Timing: A Mechanism for Monoallelic Choice During Development

**DOI:** 10.3389/fcell.2021.737681

**Published:** 2021-10-01

**Authors:** Yehudit Bergman, Itamar Simon, Howard Cedar

**Affiliations:** ^1^Department of Developmental Biology and Cancer Research, Hebrew University Hadassah Medical School, Jerusalem, Israel; ^2^Department of Microbiology and Molecular Genetics, Hebrew University Hadassah Medical School, The Institute for Medical Research Israel-Canada (IMRIC), Jerusalem, Israel

**Keywords:** epigenetic regulation, chromatin accessibility, embryonal stem cells, DNA replication, genomic imprinting, X-chromosome inactivation

## Abstract

Developmental programming is carried out by a sequence of molecular choices that epigenetically mark the genome to generate the stable cell types which make up the total organism. A number of important processes, such as genomic imprinting, selection of immune or olfactory receptors, and X-chromosome inactivation in females are dependent on the ability to stably choose one single allele in each cell. In this perspective, we propose that asynchronous replication timing (ASRT) serves as the basis for a sophisticated universal mechanism for mediating and maintaining these decisions.

## Monoallelic Expression

Although the mammalian genome has a diploid composition, many genes are regulated in a monoallelic manner. The most common form of this phenomenon is characterized by skewed allelic expression with some cells exhibiting preferential transcription for the paternal allele, some favoring the maternal allele, while other cells in this same population express this gene either biallelically or not at all (Guo et al., 2005; Savol et al., 2017; Branciamore et al., 2018; Galupa and Heard, 2018). A second type of monoallelic expression (MAE) is characterized by defined regions of the genome that are actually developmentally programmed to choose between the two alleles on the basis of stable differential marks. A classic example of this phenomenon is genomic imprinting, where a single parental allele, either the maternal or paternal, is programmed by the gametes to be transcribed in somatic cells of the offspring, while the other allele is silent. This group includes many genes, such as *Igf2* and *Snrpn*, which appear to play some role in early embryonic growth control and have been found to be involved in a number of genetic diseases (Hanna and Kelsey, 2021).

Other genome domains exhibit a random pattern of MAE with some cells selecting the maternal allele, while others choose to express the paternal copy. These regions are enriched for receptor gene clusters involved in defining cell identity by mediating interactions between the cell and its environment. This includes many of the gene arrays that make up the foundation for the immune system, olfaction and cell positioning during development (Chess et al., 1994; Rodriguez, 2013; Levin-Klein and Bergman, 2014; Chess, 2016). One of the main features of all these developmentally programmed domains is that they replicate in an asynchronous manner, one allele being copied earlier than the other during S phase, thus providing a mark that can distinguish between the two alleles (Cedar and Bergman, 2008). A similar pattern is seen in X-chromosome inactivation in somatic cells (Avner and Heard, 2001). In this perspective, we will attempt to understand how this epigenetic process is established during development, and in this way, explain the basic mechanism underlying stable allelic choice, both imprinted and random.

## Replication Timing

Due to the large size of the mammalian genome, its replication is not only extended over time, but is apparently also carried out by an organized and carefully regulated program (Goren and Cedar, 2003; Marchal et al., 2019). One of the most outstanding aspects of this process involves temporal control, with some regions of the genome undergoing DNA replication in early S phase, while others replicate late. By labeling cells with BrdU, one can actually visualize these regions as alternating chromosomal bands, representing replication time zones with an average size of about 1 Mb that colocalize with the structurally determined G banding pattern (Hand, 1978). Strikingly, this organization is also correlated to gene expression, with housekeeping and other active genes replicating early, while heterochromatin and inactive genes largely replicate in late S (Schübeler et al., 2002; Greenberg et al., 2020). In keeping with this picture, the early zones have been found to be in a relatively accessible DNaseI sensitive configuration (Kerem et al., 1984), while the late regions have a more closed structure and are localized to nuclear lamina associated domains (LADs) (Heun et al., 2001; Vogel et al., 2007; Guelen et al., 2008; van Steensel and Belmont, 2017). Furthermore, many replication time zones are regulated in a tissue or developmental-specific pattern, replicating late in most cell types, but switching to early replication in keeping with its expression profile (Holmquist, 1987; Siefert et al., 2017). Replication timing is also correlated with many important epigenetic features within the genome architecture (Rhind and Gilbert, 2013; Reverón-Gómez et al., 2018; Escobar et al., 2019). In keeping with this, several studies have provided more direct evidence that replication timing itself plays a key role in orchestrating and maintaining epigenetic states (Zhang et al., 2002; Klein et al., 2021).

## Asynchronous Replication Timing

While most regions of the genome have a fixed replication time, with both alleles being equally recognized by the trans-acting factors that govern replication timing control, there are several categories of genes that replicate in an asynchronous manner, with one allele being marked for replication early in S phase and the other, for late replication. The most striking example of this phenomenon is the X-chromosome in female somatic cells, where one copy replicates in early S, while the other copy replicates later, as demonstrated by *in situ* S-phase-specific BrdU labeling (Latt, 1973) as well as whole genome DNA sequence analysis (Koren and McCarroll, 2014; Blumenfeld et al., 2021). In keeping with this, genes on the late chromosome are generally inactive and have a non-accessible chromatin structure, characterized by DNA-methylated promoters, as well as a variety of inactivating histone modifications and variants (Mohandas et al., 1981; Jeppesen and Turner, 1993; Heard, 2004; Żylicz and Heard, 2020; Boeren and Gribnau, 2021). The actual inactivation process in the early embryo appears to take place stochastically in each individual cell, either on the paternal or maternal X chromosome and this decision is then stably maintained through future cell divisions (Heard, 2004; Sahakyan et al., 2017). Genomically imprinted gene regions represent a second category subject to asynchronous replication timing (ASRT), as determined by FISH, but in this case, it is always the same allele that is early replicating, apparently because of predetermined epigenetic events that occur in the individual gametes (Simon et al., 1999; Goren and Cedar, 2003).

In addition to these classic examples, a large number of autosomal chromosome regions (1–2 Mb in length) have been found to replicate asynchronously. This was originally documented using fluorescence *in situ* hybridization (FISH) to visualize specific gene regions in diploid cells growing in culture (Selig et al., 1992). In this assay one can visualize both copies of any particular gene region in interphase cells. In nuclei that have not yet replicated this region, one observes two single hybridization dots, representing the two alleles. After replication and subsequent segregation, however, these loci exhibit double dots. For a large percentage of the genome, both alleles are synchronized, with all nuclei exhibiting either two single or two double signals. At some loci, however, one observes a large percentage of nuclei with one allele showing a single dot (not yet replicated) and the other having a double dot (already replicated), indicating that this region replicates asynchronously (Kitsberg et al., 1993). This FISH assay encompasses two aspects of DNA replication, differential time of DNA synthesis in S-phase, as well as the time of visual chromatid segregation, suggesting that asynchronous loci are essentially characterized by allele-differential “chromosomal replication,” with structure and segregation being an important, often dominant, part of this process (Azuara et al., 2003; Rivera-Mulia et al., 2018; Blumenfeld et al., 2021).

## Principles of Allelic Choice

Asynchronous replication timing was originally observed for select genome loci containing olfactory receptor (Chess et al., 1994) or immune system (Mostoslavsky et al., 2001) gene arrays, regions clearly associated with monoallelic behavior. In both cases, each individual cell must be able to choose one allele out of the two available in order to ensure production of only one unique receptor for presentation on the cell surface. The observation of allelic asynchrony suggested that replication timing may somehow serve as a way to distinguish between the two alleles, thereby providing a simple epigenetic mark for directing allelic choice.

Use of the *Igκ* locus as a prototype provides an excellent system for better understanding ASRT and its role in monoallelic choice. During B-cell lineage formation, each cell has an equal chance of choosing the paternal, or alternatively the maternal allele for making the *Igκ* light chain (Cedar and Bergman, 2011). It was originally postulated that the decision for which allele undergoes rearrangement is completely stochastic and is mediated in trans by nuclear protein factors, with the first allele to bind the factor undergoing rearrangement (Mostoslavsky et al., 2004). The light-chain produced from this reaction would then be capable of preventing rearrangement on the other allele through feedback inhibition. This “first come, first served” mechanism has also been proposed for other cases of allelic choice, such as that seen in early embryonic random X-chromosome inactivation (Penny et al., 1996; Mutzel and Schulz, 2020).

**FIGURE 1:**
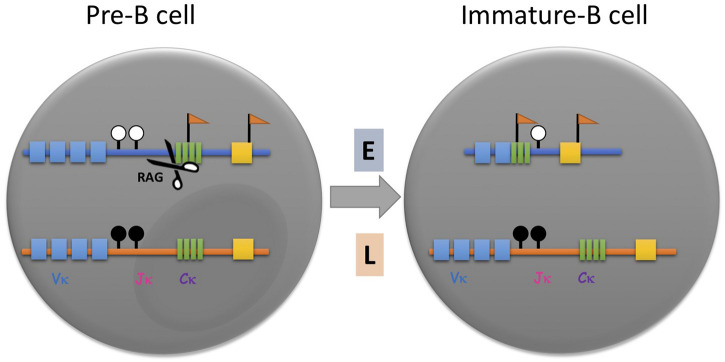
Asynchronous replication timing (ASRT) and its role in immunoglobulin allelic exclusion. In pre-B cells prior to *Igκ* rearrangement, the two unrearranged alleles, one of which replicates early (E, blue) and the other of which replicates later (L, orange), are marked with different epigenetic marks. The early replicating allele is hypomethylated at the DNA level (white lollipops) and enriched with hyperacetylated histones and methylation of histone H3 lysine 4 (flags). The late replicating allele is hypoacetylated, CpG methylated (black lollipops) and sequestered within heterochromatin. Once the pre-B cell senses a signal for rearrangement, the early replicating allele binds trans-acting factors such as B-cell-specific transcription factor, Pax5, and the rearrangement machinery (RAG), which binds to H3K4me3, thereby rendering it susceptible to rearrangement and progression to the stage of immature B cell. Thus, it is almost always the early replicating allele that undergoes rearrangement first. Vκ, variable gene segments; Jκ, joining gene segments; Cκ, constant region.

Although this trans-acting concept provided a reasonable explanation for the choosing process, FISH replication timing experiments raised the possibility that the choice of allele may actually be pre-determined, since the initial rearrangement always occurs on the early allele in mature B-cells regardless of its parental identity (Figure 1; Farago et al., 2012). Furthermore, it was demonstrated that the active allele is specifically associated with other basic structural marks, such as preferential chromatin accessibility, DNA undermethylation and localization away from the nuclear periphery, all properties that are thought to be acquired prior to the actual rearrangement step (Mostoslavsky et al., 1998, 2001; Goldmit et al., 2002, 2005; Ji et al., 2003). In pre-B cells, for example, the *Igκ* locus was found to already replicate asynchronously and when clones were prepared from single cells, it was shown by allele-specific FISH analysis that in some clones the maternal locus replicates early in every cell, while in other clones, early replication occurs consistently on the paternal allele, suggesting that the two alleles are structurally distinct from each other even prior to rearrangement. Interestingly, each clone shows an allelic pattern of chromatin accessibility and when differentiated to B-cells *in vitro*, produces the light chain antibody almost exclusively from the early allele (Figure 1; Farago et al., 2012). These experiments clearly indicate that the choosing process involves recognition of predetermined allelic marking. This same type of mechanism is probably also used for other immune system gene arrays, such as the immunoglobulin heavy chain, the T-cell receptor β locus, NK receptors as well as cytokines and their receptors, all of which have been shown to undergo asynchronous replication timing (Chess, 1998; Mostoslavsky et al., 2001; Guo et al., 2005).

Another example of allelic choice can be observed in the olfactory system where each olfactory neuron must choose a single receptor gene copy from amongst 1,000 different gene sequences that are organized as arrays within multiple asynchronous replicating domains scattered over the genome. While the choice of one specific gene sequence is apparently mediated by a single olfactosome enhancer element on chromosome 14 that can only engage one receptor gene at a time (Lomvardas et al., 2006; Markenscoff-Papadimitriou et al., 2014), there must still be a mechanism to ensure that only one of the two allelic olfactosome loci is utilized, and it is possible that this selection process is directed by its pre-existing asynchronous replication-timing mark. Interestingly, this same type of allelic non-homologous chromatin contact has also been observed at other ASRT domains, perhaps suggesting that this structure may be a general feature of allelic choice (Maass et al., 2019).

Since the FISH assay must be carried out using individual specific probes, it was previously possible to identify only a relatively small number of asynchronous replicating regions, but recent studies utilizing allelically marked hybrid pre-B cell clones have succeeded in carrying out genome-wide quantitative DNA sequence analysis of S phase cells, thus enabling the discovery of almost 150 new regions of the genome in which one allele replicates prior to the other. In each clone, some sites show early replication of the maternal allele, while others are in the opposite orientation and, in contrast to what had been observed previously, these loci are widely distributed over many different chromosomes (Blumenfeld et al., 2021). At all of these regions, the early replicating allele is preferentially more accessible (as determined by ATAC-Seq), including gene regions that are not actively expressed in these cells (e.g., olfactory receptors), suggesting that this represents an independent epigenetic mark that may be found in a wide variety of different cell types and that both ASRT and monoallelic accessible chromatin structure exist prior to expression, at a stage when it may actually be involved in the allelic choice process itself. In addition to these important structural features, genomic analysis also revealed new, potentially mono-allelic gene functions located preferentially in ASRT domains, including the taste receptors, the vomeronasal receptors, as well as chemokines and their receptors that are used for chemotaxis, all of which are organized as gene arrays (Blumenfeld et al., 2021). It is interesting in this regard that the olfactory receptor system evidently plays a dual role as an odor receptor, as well as a guiding element that directs specific neurons to their proper location in the olfactory bulb (Mombaerts et al., 1996).

## Development

A number of different studies have noted that, in general, the ASRT pattern can be detected in a wide variety of different cell types independently of whether these regions are actually expressed, thus suggesting that the establishment of ASRT must take place during very early development. This concept is also supported by the observation that all of the known ASRT loci also replicate asynchronously in early embryonic stem cells (ESCs) (Gribnau et al., 2003; Alexander et al., 2007; Blumenfeld et al., 2021).

Early studies *in vivo* were influential in elucidating the developmental timing of this process by showing that loci associated with ASRT start off in the early embryo by replicating synchronously, as observed in cells of the morula (6–16 cells), blastula (∼60 cells) and inner cell mass (ICM) (Mostoslavsky et al., 2001; Shufaro et al., 2010). Thus, the actual process of establishing this mark must occur during the transition to implantation stage. In an attempt to mimic this process *in vitro* and thereby decipher its mechanism, ES cells were converted to a more-naive pre-implantation stage by culturing them in 2i medium and this was sufficient to revert all ASRT loci to a synchronous replication pattern. Furthermore, subsequent removal of the 2i medium quickly restored these cells to their original state, with these loci already becoming asynchronous in the first division cycle after transfer (Masika et al., 2017).

When analyzed in detail, this ASRT initiation event turns out to be very interesting, since for each individual ASRT locus it is always one specific parental allele that is chosen to be early during the first round. For some sites, the paternal allele is set up as early, while for other sites, it is the maternal allele that replicates early and this serves to establish a fixed coordinated pattern of parallel and anti-parallel ASRT loci across the genome (Masika et al., 2017; Blumenfeld et al., 2021). This pre-determined pattern indicates that at every ASRT domain, each parental allele must already be marked in the gamete in a manner that will allow it to dictate whether to undergo early or late replication during the first cycle of ASRT at the time of implantation. Thus, the information for distinguishing between the alleles is inherently encoded by epigenetic tags derived from the individual homozygous gametes and thus does not actually involve making a stochastic decision between two equal alleles. Although the identity of these marks is not known, ES cells lacking all DNA methylation were unable to generate this asynchrony, suggesting that this early marking process may, in some way, involve DNA methylation (Masika et al., 2017) in conjunction with histone marks, as has been shown to be the case for genomic imprinting (Nakamura et al., 2007).

**FIGURE 2:**
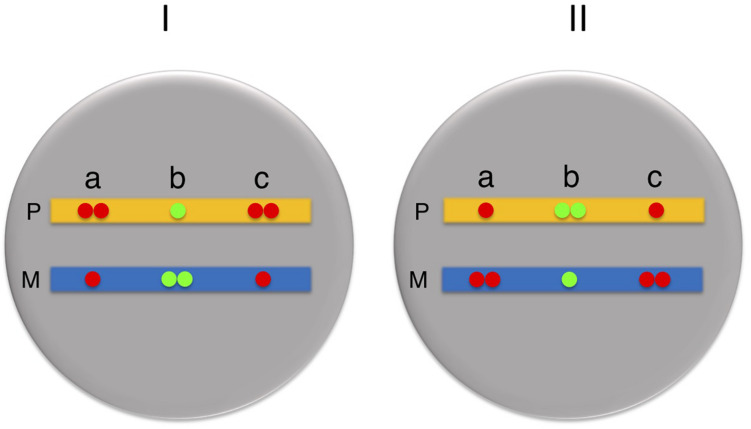
Orientation of asynchronous regions. All cells in the body are in one of two mirror-image states (I or II) with regard to their Paternal (P, yellow) and Maternal (M, blue) alleles. Probes a and c always replicate in a parallel manner, with both being early on the same allele, while probes a and b always replicate in an antiparallel manner both in cell I and cell II.

Once this initial orientation pattern is established, subsequent cell divisions still perpetuate the asynchronous state, but each cycle is then subject to allele switching, so that all loci set up as maternal early will generate daughter cells characterized by paternal early replication, while all loci generated as paternal early will undergo a complete switch to maternal early (Masika et al., 2017). This automatic switching behavior essentially preserves the original parallel or antiparallel relationship between the many ASRT loci in the genome and thus sets up a bimodal population containing two “enantiomeric” cell types, each having a mirror image ASRT orientation profile (Figure 2; Blumenfeld et al., 2021). Switching continues throughout early stem-cell-like stages, until commitment comes into play at the time of definitive differentiation when cells then begin to clonally maintain each “enantiomer” separately. In line with this notion, it is likely that all tissues in the body are constituted from a mixture of two mirror image ASRT states.

During lymphoid development in the immune system, for example, hematopoietic stem cells (HSCs) and multi-potent precursor cells (MPPs) are still in the allele switching mode, but progression to Common Lymphoid Precursors (CLPs) is accompanied by commitment to one specific direction, which is then clonally maintained during subsequent stages of lineage commitment (Farago et al., 2012). Taken together, these findings indicate that switching represents a form of plasticity that preserves the potential for stem cells to use either one of two fixed options. Upon differentiation, they lose this plasticity and become committed to targeting one allele. In the immune system, it is this allelic clonality that actually allows the formation of memory cells with the potential to mount an antibody defense to specific antigens.

## Mechanisms of Allelic Choice

The classical way of thinking about “allelic choice” usually entails interactions between trans-acting factors in the nucleus and identical cis-acting sequences that compete with one another. This model assumes that both alleles have the same probability of engagement, making it difficult to choose only one and then maintain this decision for extended periods of time. Kinetically, this type of mechanism would also require low concentrations of the trans-acting protein factor, perhaps combined with a feedback regulatory loop that can quickly prevent the other allele from being activated, a pathway which has been shown to exist in the immune system (Coleclough, 1983; Yancopoulos and Alt, 1986; Gorman and Alt, 1998; Liang et al., 2004). Probability considerations predict that low concentrations of the activating factor could indeed bring about targeting of one allele prior to the other, but this model also predicts that in many cells, neither allele may get activated, a situation which is not appropriate for carrying out programmed developmental decisions.

Early developmental programming of ASRT represents an excellent alternative solution to the problem of choosing, by providing a stable epigenetic mark in cis that distinguishes between two almost-identical alleles in the same cell, making one more accessible than the other. This structural difference is set up early in the embryo and then maintained in all cell types where it can provide a template for preferentially activating one allele as opposed to the other. Thus, the choice of allele is a built-in part of replication-time-directed genome structure (Klein et al., 2021), waiting to be utilized in a specific manner in individual cell types. There is thus no need for “choosing” in trans over and over again in each cell, since the decision process itself has already been pre-coded in cis in all somatic cells. This may be accomplished in a very simple and, in fact, fool-proof manner by pre-marking each allele separately in the gametes (i.e., in cells carrying only one allele) by an, as yet, unknown mechanism. This information is then employed to set up differential asynchronous replication in the implantation embryo.

**FIGURE 3:**
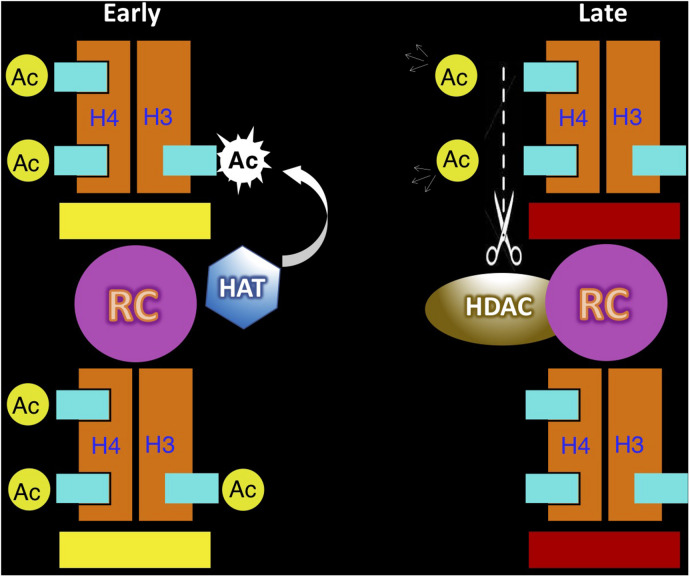
Autonomous maintenance of asynchronous replication timing (ASRT). This model demonstrates how histone acetylation (Ac) at the replication origin may serve as a mark for the autonomous maintenance of replication timing on individual alleles. In cells set up to replicate one allele early (yellow bar) and the other late (red bar), the early allele is marked by acetylation of both histone H3 and H4 at the replication origin, while the late replicating allele lacks histone acetylation. During the next cycle the acetylated allele (left) will be recognized to undergo replication in early S, thus generating two copies of the DNA template, with the original nucleosome remaining on one copy **(bottom)** and a newly made nucleosome **(top)** placed on the other. Since histone H4 becomes acetylated immediately after its synthesis in the cytoplasm, this new nucleosome is already acetylated at these sites. Acetylation of histone H3 is carried out by a histone acetylase (HAT) that is associated with the replication machinery, but only in early S phase. Thus, following early replication the origin on both chromatids is now packaged with nucleosomes marked for early replication in the next cell cycle. The other allele in the same cell (right) is marked for late replication at the origin and is packaged with nucleosomes lacking histone acetylation. This allele is recognized for replication as the cell passes through early S phase, but will then undergoes replication during late S. The original nucleosome will remain with one of two DNA copies (bottom), while the other DNA template will get packaged with a new nucleosome, already acetylated on histone H4. During late S, the replication complex (RC) contains a cell-cycle-dependent histone deacetylase (HDAC) that can remove these H4 acetyl groups, thus guaranteeing that both chromatids will be packaged with un-acetylated histones and effectively regenerate the mark that directs late replication in the next cell cycle. It should be noted that this mechanism may also be able to accommodate the stem-cell switching mode of ASRT by automatically switching the acetylation state on each allele at the end of S phase in every cell cycle. As opposed to all other mechanisms for epigenetic maintenance which are carried out by “copying” specific marks, replication timing memory is time-based and takes advantage of differential cell-cycle properties.

It is still a mystery how the asynchronous replication state can be maintained through cell division and replication. Unlike most structural features defined by fixed epigenetic marks such as DNA methylation, maintenance of ASRT is complicated by the fact that this property can exist in either a switching or committed mode. For this reason, we suggest that replication timing may itself be an epigenetic feature that has an inherent mechanism that allows it to be autonomously perpetuated. A great deal of evidence indicates that the time of replication for each locus is set up during the G1 stage of the cell cycle and this is accomplished by the recruitment of protein complexes at all the coordinated replication origins in a given time zone (Goren and Cedar, 2003). This marking system provides information that determines the time of replication in S. In the case of ASRT, one allele is marked for early replication and the other for late. Since S-phase progression is associated with programmed changes in nuclear environment, each allele will encounter a different set of trans-acting factors, which may then mark the newly replicated allele as having been copied in either early or late S. A major candidate for this type of regulation is histone H3/H4 acetylation, which has already been shown to modify nucleosomes at the replication fork in an S-phase specific manner (Zhang et al., 2002; Lande-Diner et al., 2009). Following replication and cell division, this temporal-dependent feature can then be used for re-establishing the allele-specific time of replication during the next cell cycle, thus maintaining ASRT (Figure 3).

It should be noted that in all of the developmental systems where ASRT may play a role in allelic choice, ASRT does not seem to be a key element in the process of gene inactivation or activation itself, with this being accomplished by a variety of many different mechanisms that may include DNA methylation, histone modification, ncRNA, and others (Żylicz and Heard, 2020; Boeren and Gribnau, 2021). ASRT would simply serve as a means to mark the two alleles differently, thus enabling these other factors to operate on only one of the two copies. From this perspective, the underlying function of ASRT is the process of “allelic choice” itself.

## Biological Function of ASRT-Based Monoallelic Expression

In order to put ASRT into a more biological perspective, it is worthwhile considering the potential functions of monoallelic choice and the possible molecular mechanisms that could mediate this process. From a careful analysis of the genes located in asynchronously replicating domains, it emerges that many of these regions include gene arrays, each of which contain a variety of alternate receptor genes that make up a reservoir from which each cell can uniquely choose one for presentation on the cell surface. Because the genome is diploid, this process would not only require the stochastic selection of a single receptor gene within an array, but may also be dependent on a reliable mechanism for ensuring that only a single one of the two alleles is actually activated for transcription and it is likely that this choice is mediated by the ASRT-associated chromosomal and nuclear structural features that essentially make these loci epigenetically “monoploid.” Indeed, because ASRT operates at the regional as opposed to local level, it is capable of carrying out a form of epigenetic regulation that is uniquely appropriate for controlling large gene arrays. Taken together, this developmental system provides a mechanism for the stable and reliable programming of choices within the immune, sensory and motility systems by defining cell identity.

## Holistic Model

Allelic choice by means of asynchronous replication timing may represent a subset of general strategies that utilize genomic imprinting. Extensive research on the mechanisms involved in imprinting have indicated that DNA methylation plays a prominent role by marking gene sequences in one of the gametes, thereby designating this allele as being inactive (Li et al., 1993). Because DNA methylation can be maintained autonomously at every cell division (Cedar and Bergman, 2012), this early generated mark is then remembered in cis throughout development, thereby perpetuating a decision that was initially made at a stage when both alleles were completely separated from each other. Independently of being epigenetically marked by DNA methylation (Gribnau et al., 2003), imprinted genes are clustered within asynchronous replication timing domains (May et al., 2008; Shufaro et al., 2010), with all tested cases showing a paternal early pattern (Simon et al., 1999), regardless of their expression profile. Another example of non-random allelic silencing is the paternal specific X-chromosome inactivation that takes place in extraembryonic tissues of the female mammal and in all cell types of marsupials (Migeon et al., 1989; Samollow et al., 1995). In the mouse, it has been demonstrated that this choice is associated with differential early replication of the paternal allele (Takagi and Sasaki, 1975).

**FIGURE 4:**
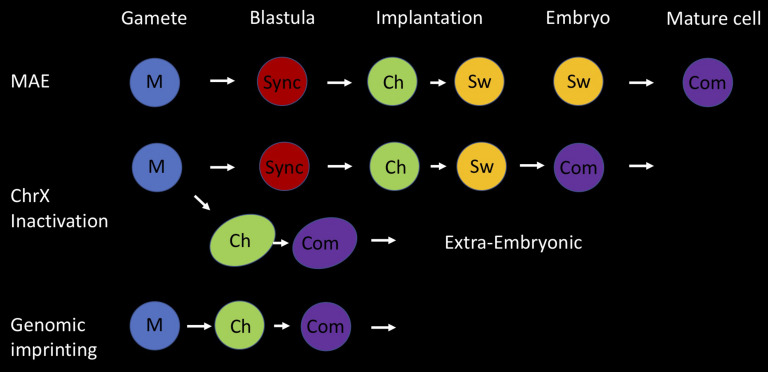
Holistic model for asynchronous replication timing (ASRT). Three different forms of developmentally based monoallelic expression (MAE) are associated with regional asynchronous replication timing; genomic imprinting, inactivation of one X chromosome in female animals and random MAE of autosomal genes. Here we present an integrative developmental model for the establishment of allelic choice with its basic building blocks. This process is accomplished in three seminal steps; marking (M), choice (Ch), and commitment (Com). In all cases, the two alleles are marked (M) differentially in the gametes, where each parental allele is separated from its partner. One is marked as being of maternal origin and the other as being of paternal origin. For random MAE, the two alleles replicate synchronously (sync) in cells of the early pre-implantation embryo. Asynchronous replication is initiated at the stage of implantation, when one allele is chosen (Ch) to replicate early and the other late, based on the epigenetic mark (M) derived from the gametes. In all subsequent replication cycles, the two alleles switch (Sw) their time of replication in S phase, but each locus still retains its parallel or anti-parallel orientation relative to other ASRT loci in the genome. Switching continues in cells of the embryo until they commit (Com) to one fixed parental replication pattern. Imprinted gene regions are initially marked in the gametes, but immediately adopt allele-specific asynchronous replication timing in the early embryo and become committed (Com) to this fixed pattern in all cells, without going through a stage of switching. The inactivation of one copy of ChrX in female embryos utilizes both these pathways. After marking in the gametes, they establish an imprinted pattern of ASRT during formation of the extra-embryonic tissues, while they replicate synchronously in embryonic cells before setting up ASRT at the time of implantation and then undergoing switching to enable random X inactivation and commitment, post implantation.

It appears that random asynchronous replication timing and its association with monoallelic choice has many of the features associated with genomic imprinting (Figure 4). In both of these basic processes, regulation occurs at a regional level, involves allele differential replication and is faithfully maintained throughout development. As opposed to imprinting, which has a fixed parental orientation pattern, random ASRT allows the selection of either the maternal or paternal allele, but the strategy used to initially establish the differential state is essentially very similar in that it involves an early developmental choice of one fixed allele to be early replicating. The decision itself is actually initiated in the individual gametes, at a stage where there is only one allele, which is then epigenetically marked to dictate either early or late replication when ASRT is set up in the early embryo. This mechanism thus provides a simple and sophisticated system for avoiding having to choose between two identical alleles in a single cell. Following this initial step, the only difference between random ASRT and imprinting is the subsequent introduction of a switching mechanism, making it possible to get exclusive expression from either the maternal or paternal allele.

It is worthwhile noting that random X-inactivation in female embryos also occurs at about the time of implantation and generates some cells in which the maternal X is inactivated and others in which it is the paternal, with the inactive chromosome always being differentially late replicating (Mlynarczyk-Evans et al., 2006). It is very possible that this process is also part of the random allelic choice system that occurs on select autosomal regions. In keeping with this idea, it has been demonstrated that the two X-chromosomes actually replicate asynchronously in ES cells with an allelic pattern that is not preserved in single-cell clones, suggesting that this entire chromosome may be subject to allelic switching similar to what occurs in autosomal ASRT, thus explaining how one X in each cell is chosen for inactivation in the early embryo (Gribnau et al., 2005; Mlynarczyk-Evans et al., 2006).

Taken together, this suggests that X-chromosome inactivation constitutes a general prototype for both random and non-random allelic choice (Figure 4). One allele, the paternal, is initially marked for early replication and retains this memory to set up imprinted X inactivation in cells destined for the formation of extra-embryonic tissues. Further pre-implantation embryonic stages become subject to switching, which then serves as the basis for random inactivation that, in this case, becomes clonally committed shorty after implantation. Although the main role of ASRT on the X-chromosome presumably involves dosage compensation, it should be noted that at least at one locus this mechanism serves the more general function of defining single-cell specificity. The red and green pigment genes for color vision located in a small array on chromosome X are individually activated by a common enhancer sequence that can only choose one at a time (Wang et al., 1992). In males, where there is only a single X chromosome, this decision allows the generation of unique pigment cells (either red or green) in the retinal cone. In females, however, where there are two X-chromosomes, the production of two different pigments in the same cell is prevented by X-inactivation, in a manner that is very similar to the function of ASRT in many autosomal loci.

Monoallelic expression appears to constitute a fundamental aspect of mammalian biology and development, which by its very nature must utilize epigenetic regulation. In this perspective, we have proposed that asynchronous replication timing plays a unique role in the establishment and maintenance of allelic choice. Specific regions in the genome become differentially marked in the individual gametes and this feature is then used in the embryo as a blueprint for setting up structural allelic differences that are maintained in all cells of the body, where it can, if needed, enable allelic choice. Because this system is essentially based on preserving a “difference” between the alleles with an option to switch their identity, it can serve as a mechanism for both genomic imprinting as well as random MAE, processes that underlie both dosage compensation as well as the determination of cell identity (Figure 4).

## Author Contributions

All authors listed have made a substantial, direct and intellectual contribution to the work, and approved it for publication.

## Conflict of Interest

The authors declare that the research was conducted in the absence of any commercial or financial relationships that could be construed as a potential conflict of interest.

## Publisher’s Note

All claims expressed in this article are solely those of the authors and do not necessarily represent those of their affiliated organizations, or those of the publisher, the editors and the reviewers. Any product that may be evaluated in this article, or claim that may be made by its manufacturer, is not guaranteed or endorsed by the publisher.
